# Negative binomial mixed models for analyzing microbiome count data

**DOI:** 10.1186/s12859-016-1441-7

**Published:** 2017-01-03

**Authors:** Xinyan Zhang, Himel Mallick, Zaixiang Tang, Lei Zhang, Xiangqin Cui, Andrew K. Benson, Nengjun Yi

**Affiliations:** 1 Department of Biostatistics, University of Alabama at Birmingham, Birmingham, AL 35294-0022 USA; 2 Department of Biostatistics, Harvard T.H. Chan School of Public Health, Boston, MA 02115 USA; 3 Program in Medical and Population Genetics, the Broad Institute, Cambridge, MA 02142 USA; 4 Department of Biostatistics, School of Public Health, Medical College of Soochow University, Suzhou, 215123 China; 5 Department of Food Science and Technology and Core for Applied Genomics and Ecology, University of Nebraska, Lincoln, NE 68583 USA

**Keywords:** Count data, Correlated measures, Microbiome, Metagenomics, Random effects, Negative binomial model, Penalized Quasi-likelihood

## Abstract

**Background:**

Recent advances in next-generation sequencing (NGS) technology enable researchers to collect a large volume of metagenomic sequencing data. These data provide valuable resources for investigating interactions between the microbiome and host environmental/clinical factors. In addition to the well-known properties of microbiome count measurements, for example, varied total sequence reads across samples, over-dispersion and zero-inflation, microbiome studies usually collect samples with hierarchical structures, which introduce correlation among the samples and thus further complicate the analysis and interpretation of microbiome count data.

**Results:**

In this article, we propose negative binomial mixed models (NBMMs) for detecting the association between the microbiome and host environmental/clinical factors for correlated microbiome count data. Although having not dealt with zero-inflation, the proposed mixed-effects models account for correlation among the samples by incorporating random effects into the commonly used fixed-effects negative binomial model, and can efficiently handle over-dispersion and varying total reads. We have developed a flexible and efficient IWLS (Iterative Weighted Least Squares) algorithm to fit the proposed NBMMs by taking advantage of the standard procedure for fitting the linear mixed models.

**Conclusions:**

We evaluate and demonstrate the proposed method via extensive simulation studies and the application to mouse gut microbiome data. The results show that the proposed method has desirable properties and outperform the previously used methods in terms of both empirical power and Type I error. The method has been incorporated into the freely available R package BhGLM (http://www.ssg.uab.edu/bhglm/ and http://github.com/abbyyan3/BhGLM), providing a useful tool for analyzing microbiome data.

## Background

The advent of next-generation sequencing (NGS) technology enables the generation of large volume of metagenomic sequencing data at moderate cost [1–3]. This opens a new era of metagenomics studies to explore microbial communities sampled directly from the environments without need for cultivation [4–6]. The metagenomic sequencing data provide valuable resources for investigating associations between the microbiome and host environmental/clinical factors. Accurately identifying and understanding these associations is critical to elucidate the true roles of the microbiome in health and disease states and for development of new diagnostics and therapeutic targets based on the microbiome [7–10]. Recent studies have found that the human microbiome is influenced by various host factors including genotype [11–14], lifestyle such as dietary habit [15, 16], physiological status such as aging [17], pathophysiological status [18], and host environment [19]. Abnormalities in compositional features of the microbiome are associated with human diseases such as obesity [20], diabetes [21], inflammatory bowel disease [22], and cancers [23].

Despite our ability to generate large-scale metagenomic sequencing data, study of the microbiome is still in its infancy and many challenges exist to decipher the mechanisms through which the microbiome affects human health. One of the challenges is the development of robust and powerful statistical methods and computational tools for properly analyzing and interpreting complex microbiome data. High-throughput microbiome datasets generated by the 16S ribosome RNA (rRNA) gene sequencing or shotgun metagenomic sequencing have some properties that require tailored analytic tools; these include count compositional structure, varied total sequence reads across samples, over-dispersion and zero-inflation. Several methods have been developed to tackle these properties. One way to account for varing total reads is normalization, i.e., conversion of the sequence counts to the relative abundance (or proportion) using the total sum, mean, or median of representative OTUs across all samples [7, 24–27]. The negative binomial regression, which is a standard statistical method for analyzing over-dispersed count observations, has been recently applied to microbiome data [28]. On the other hand, several zero-inflated models have also been proposed to correct for excess zero counts in microbiome measurements, including zero-inflated Gaussian, lognormal, negative bimomial and beta models [25, 29–32].

In addition to the challenges resulting from the characteristics of microbiome count data, there are other statistical issues due to the study designs commonly used in microbiome studies. Microbiome studies ususally collect samples from study designs that bring about hierarchical, spatial, and temporal dependences [32–39], which introduce correlation among the samples and thus further complicate the analysis and interpretation of microbiome count data. Since related samples tend to harbor more similar microbiota than unrelated ones [11, 38], ignoring the correlation among samples can result in biased inference and misleading results. Thus, statistical models for accounting for the correlation among samples are crucially required [11, 38, 40].

The literature on mixed-effects models for analyzing microbiome count data is sparse. Most of the previous studies resort to linear mixed models (LMMs) to account for hierarchical structures in microbiome study designs by treating transformed data as normally distributed responses [33–35, 37, 39]. Such methods may be suboptimal due to the discrete and compositional nature of the microbiome measures and can be hard to interpret on the original scale, which might lead to challenges in future prediction tasks and replication studies. To address these limitations, we propose negative binomial mixed models (NBMMs) for directly modeling the raw microbiome count data, which bypasses the need for transformation. Although not dealing with zero-inflation, the proposed mixed-effects models not only efficiently handle over-dispersion and varying total reads, but also account for correlation among the samples. We develop a flexible IWLS (Iterative Weighted Least Squares) algorithm to fit the proposed NBMMs by taking advantage of the standard procedure for fitting linear mixed models. Through extensive simulations, we show that the NBMMs outperform the negative binomial model and the previously used linear mixed models in terms of empirical power and false positive rates. We also apply our method to previously published mouse gut microbiome data to detect taxa significantly associated with high-fat diet. The proposed method is capable of identifying biologically significant taxa, consistent with the existing literature. We have implemented the method in the freely available R package BhGLM, providing a useful tool for microbiome studies.

## Methods

### Negative Binomial Mixed Models (NBMMs) for microbiome studies

Typical microbiome data generated by the 16S rRNA gene sequencing or the shotgun metagenomic sequencing consist of the following components (see Table 1): 1) **Counts**, *C*_*ij*_, for *n* samples and *m* features. The features may refer to bacterial taxa at different hierarchical levels (species, genus, classes, etc.), groups of correlated taxa, gene functions, or pathways, etc.; 2) **Total sequence read** (also referred to as depths of coverage or library size), *T*_*i*_, for each sample; 3) **Host factors**, *X*_*i*_, representing host clinical/environmental or genetic variables; 4) **Sample variables**, *Z*_*i*_, representing sample collection identifier in the hierarchical study design, such as family structure, repeated measures from multiple body sites or time points. The goal is to detect associations between microbiome features *C*_*ij*_ and host factors *X*_*i*_. The total sequence reads vary from sample to sample by orders of magnitude and can largely bias comparison of counts across samples, and thus should be accounted for in the analysis. Sample variables *Z*_*i*_ introduce hierarchical, spatial, and temporal dependence of microbiome counts, and should be included in the analysis as random factors.

**Table 1:** Microbiome Data Structure

	Feature 1	Feature 2	*· · ·*	Feature *m*	Total read	Host factors	Sample variables
Sample 1	*C*_*11*_	*C*_*12*_	*· · ·*	*C*_*1m*_	*T*_*1*_	*X*_*1*_	*Z*_*1*_
Sample 2	*C*_*21*_	*C*_*22*_	*· · ·*	*C*_*2m*_	*T*_*2*_	*X*_*2*_	*Z*_*2*_
·	·	·	·	·	·	·	·
·	·	·	·	·	·	·	·
·	·	·	·	·	·	·	·
Sample *n*	*C*_*n1*_	*C*_*n2*_	*· · ·*	*C*_*nm*_	*T*_*n*_	*X*_*n*_	*Z*_*n*_

Similar to most existing methods, we separately analyze each feature (count response) in a univariate fashion. For notational simplification, we denote *y*_*i*_ = *C*_*ij*_ for any given feature *j*. We assume that the count response *y*_*i*_ follows the negative binomial distribution:

1 $$ {y}_i\sim NB\left({y}_i\Big|{\mu}_i,\theta \right)=\frac{\varGamma \left({y}_i+\theta \right)}{\varGamma \left(\theta \right){y}_i!}\cdot {\left(\frac{\theta }{\mu_i+\theta}\right)}^{\theta}\cdot {\left(\frac{\mu_i}{\mu_i+\theta}\right)}^{y_i} $$

where *μ*_*i*_ and *θ* are the mean and the shape parameter, respectively, and Γ(·) is the gamma function. The negative binomial distribution can be expressed as a gamma mixture of Poisson distribution [41]: *y*_*i*_ ~ Poisson(*y*_*i*_|*μ*_*i*_ *ε*_*i*_) and *ε*_*i*_ ~ Gamma(*θ*, *θ*). It can be derived that E(*y*_*i*_) = *μ*_*i*_, $\mathrm{V}\mathrm{a}\mathrm{r}\left({y}_i\right)={\mu}_i+\frac{\mu_i^2}{\theta }$, and Var(*y*_*i*_) ≥ E(*y*_*i*_). Thus, the shape parameter *θ* controls the amount of over-dispersion. When *θ* = +∞, Var(*y*_*i*_) = *μ*_*i*_ and the negative binomial model converges to a Poisson model that cannot deal with over-dispersion.

Our negative binomial mixed models (NBMMs) relate the mean parameters *μ*_*i*_ to the host factors *X*_*i*_ (including the intercept), the sample variables *Z*_*i*_ and the total sequence reads *T*_*i*_ via the link function logarithm:

2 $$ \log \left({\mu}_i\right)= \log \left({T}_i\right)+{X}_i\beta +{Z}_ib $$

where log(*T*_*i*_) is the offset, which corrects for the variation of the total sequence reads across the samples, *β* is the vector of fixed effects for the host factors *X*_*i*_, and *b* is the vector of *K* random effects for the sample variables *Z*_*i*_. The random effects are used to model the correlation among the samples and the multiple sources of variation, and thus to avoid biased inference on the effects of the host factors *X*_*i*_. The vector of the random effects is usually assumed to follow the multivariate normal distribution [42, 43]:

3 $$ b\sim {N}_K\left(0,\varPsi \right) $$

where Ψ is a positive-definite variance-covariance matrix that determines the form and complexity of random effects. Although in principle our NBMMs can deal with various patterns of Ψ, we here describe the method with a simple case where the random effects are independent, i.e., *b* ~ *N*_*K*_(0, *τ* ^2^ *I*).

### The IWLS algorithm for fitting the NBMMs

We propose an IWLS (Iterative Weighted Least Squares) algorithm to fit the NBMMs by extending the commonly used algorithms for fitting generalized linear models (GLMs) and generalized linear mixed models (GLMMs). For any fixed shape parameter *θ*, the negative binomial density is of the exponential form, $NB\left({y}_i\Big|{\mu}_i,\theta \right)= \exp \left\{\frac{y_i{\vartheta}_i-b\left({\vartheta}_i\right)}{\phi }+c\left({y}_i,\phi \right)\right\}$, where ${\vartheta}_i= \log \frac{\mu_i}{\mu_i+\theta }$, *ϕ* = 1, $b\left({\vartheta}_i\right)=-\theta \log \left(1-{e}^{\log \frac{\mu_i}{\mu_i+\theta }}\right)=-\theta \log \left(1-{e}^{\vartheta_i}\right)$, and $c\left({y}_i,\phi \right)= \log \left(\frac{\varGamma \left({y}_i+\theta \right){\theta}^{\theta }}{\varGamma \left(\theta \right){y}_i!}\right)$. Therefore, the negative binomial model is a special case of generalized linear models (GLMs) for any fixed *θ*. If *θ* is an unknown parameter, the negative binomial model is not a GLM. However, the NBMMs can be fit by iteratively updating the parameters (*β*, *b*, *τ* ^2^) and *θ*. Conditional on *θ*, the NBMM is a special GLMM and thus the parameters (*β*, *b*, *τ* ^2^) can be updated by using the GLMMs procedure. Conditional on (*β*, *b*), the shape parameter *θ* can be updated by maximizing the NB likelihood using the standard Newton–Raphson algorithm [44].

Conditional on *θ*, we update the parameters (*β*, *b*, *τ* ^2^) by extending the IWLS algorithm or equivalently the Penalized Quasi-Likelihood procedure for fitting GLMMs. [42, 44–46] The IWLS algorithm proceeds to approximate the generalized linear model likelihood by a weighted normal likelihood and then update the parameters from the weighted normal model [41, 47]. Conditional on the shape parameter *θ*, the fixed effects *β* and the random effects *b*, the negative binomial likelihood *NB*(*y*_*i*_|*μ*_*i*_, *θ*) can be approximated by the weighted normal likelihood:

4 $$ NB\left({y}_i\Big|{\mu}_i,\theta \right)\approx N\left({t}_i\Big|{\eta}_i,\kern0.5em {w}_i^{-1}\right) $$

where *η*_*i*_ = log(*T*_*i*_) + *X*_*i*_ *β* + *Z*_*i*_ *b*, the ‘normal response data’ *t*_*i*_ and the ‘weights’ *w*_*i*_ are called the pseudo-response and the pseudo-weights, respectively. The pseudo-response *t*_*i*_ and pseudo-weights *w*_*i*_ are calculated by:

5 $$ {t}_i={\widehat{\eta}}_i-\frac{L\hbox{'}\left({y}_i\Big|{\widehat{\eta}}_i,\widehat{\theta}\right)}{L\hbox{'}\hbox{'}\left({y}_i\Big|{\widehat{\eta}}_i,\widehat{\theta}\right)},\kern0.5em \mathrm{and}\kern0.5em {w}_i=-L\hbox{'}\hbox{'}\left({y}_i\Big|{\widehat{\eta}}_i,\widehat{\theta}\right) $$

where ${\widehat{\eta}}_i= \log \left({T}_i\right)+{X}_i\widehat{\beta}+{Z}_i\widehat{b}$, $L\left({y}_i\Big|{\widehat{\eta}}_i,\widehat{\theta}\right)= \log NB\left({y}_i\Big|{\widehat{\mu}}_i,\widehat{\theta}\right)$, *L* ' (*y*_*i*_|*η*_*i*_, *θ*) = *dL*(*y*_*i*_|*η*_*i*_, *θ*)/*dη*_*i*_, *L* '' (*y*_*i*_|*η*_*i*_, *θ*) = *d* ^2^ *L*(*y*_*i*_|*η*_*i*_, *θ*)/*dη*_*i*_^2^, and $\left(\widehat{\beta},\widehat{b}\right)$ and $\widehat{\theta}$ are the current estimates of (*β*, *b*) and *θ*, respectively. Therefore, the NBMMs can be approximated by the linear mixed model with *w*_*i*_ as weights:

6 $$ {t}_i= \log \left({T}_i\right)+{X}_i\beta +{Z}_ib+{w}_i^{-1/2}{e}_i,\kern0.5em b\sim {N}_K\left(0,{\tau}^2\right),\kern0.5em e\sim {N}_n\left(0,{\sigma}^2I\right) $$

The parameters (*β*, *b*, *τ* ^2^, σ^2^) are then updated from this linear mixed model by using the standard algorithm for fitting LMMs.

In summary, the IWLS for fitting the NBMMs is an iterative algorithm and proceeds as follows:

1. Initialize *β*, *b*, and *θ* some plausible values;
2. For *j* = 1, 2, · · · :
  - Based on the current values (*β* ^(*j* − 1)^, *b* ^(*j* − 1)^, *θ* ^(*j* − 1)^), calculate pseudo-response *t*_*i*_^(*j*)^ and pseudo-weights *w*_*i*_^(*j*)^;
  - Update (*β*, *b*, *τ* ^2^, σ^2^) by fitting the LMM (6);
  - Update *θ* by the standard Newton–Raphson algorithm.
3. Repeat Step 2) until convergence.

We use the criterion (*η* ^(*j*)^ − *η* ^(*j* − 1)^)^2^ < *ε*(*η* ^(*j*)^)^2^ to assess convergence, where ${\eta}^{(j)}={\displaystyle \sum_{i=1}^n\left( \log \left({T}_i\right)+{X}_i{\beta}^{(j)}+{Z}_i{b}^{(j)}\right)}$, and *ε* is a small value (say 10^−5^). At convergence of the algorithm, we get the maximum likelihood estimates of the fixed effects *β*_*k*_ and their confidence intervals from the final LMM. We then can test H_0_: *β*_*k*_ = 0 following the LMMs framework.

It has been noted that the maximum likelihood estimator of the shape parameter *θ* in negative binomial models often lacks robustness and may be severely biased or even fail to converge especially if the sample size is small [48]. Similar to quasi-GLMs [47] and GLMMs [44–46], the above IWLS algorithm for fitting the NBMMs introduces an additional parameter *σ* ^2^, which can correct for over-dispersion to some extent even if *θ* is not well estimated. Therefore, our approach can be robust and efficient to deal with over-dispersed microbiome count data.

### Computer software for implementing the proposed method

We have created an R function glmm for setting up and fitting the NBMMs. The function glmm works by repeated calls to the function lme in the package **nlme**. The function lme is widely used for analyzing linear mixed models. The function glmm takes advantage of the nice features in lme, and thus provides an efficient and flexible tool for analyzing microbiome count data. We have incorporated the function glmm into our R package **BhGLM**, which is freely available from the website http://www.ssg.uab.edu/bhglm/ and the public GitHub repository http://github.com/abbyyan3/BhGLM that includes R codes for examples, simulation studies and real data analysis in this article.

## Results

### Simulation studies

#### Simulation design

We used simulation studies to assess the performance of the proposed method and to better understand the properties of our procedure. Several studies have recently performed simulations for microbiome data [25, 29, 30, 49, 50], most of which use negative binomial distributions to generate microbiome counts. We followed the simulation framework of Sohn et al. [30] to simulate microbiome counts from negative binomial distributions and extended their framework to include random effects and correlation structures:

$$ {y}_i\sim NB\left({y}_i\Big|{\mu}_i,\theta \right),\kern0.5em  \log \left({\mu}_i\right)= \log \left({T}_i\right)+\mu +{x}_i\beta +{z}_ib,\kern0.5em b\sim {N}_K\left(0,{\tau}^2I\right),\kern0.5em i=1,\cdots, n $$

We simulated *n* = 200 and 400 individuals clustered into *K* = *n*/10 groups (e.g., families), respectively. We considered a binary fixed-effect variable *x*_*i*_ and a random-effect factor *z*_*i*_. The random-effect factor *z*_*i*_ was a multinomial variable, i.e., *z*_*i*_ = (*z*_*i*1_, ⋯, *z*_*iK*_), z_*ij*_ = 0 or 1, ∑_*j* = 1_^*K*^ *z*_*ij*_ = 1, which assigned *n* samples into *K* groups and introduced correlation for the samples within a same group. To simulate *x*_*i*_ and *z*_*i*_, we first generated two continuous variables from the standard normal density N(0, 1) with a preset correlation coefficient *ρ*, and then transformed the first continuous variable to a binary indictor *x*_*i*_ based on the quantile of 40% and the second continuous variable to a multinomial variable *z*_*i*_ based on the *K* quantiles. Our goal was to evaluate the performance of the proposed method for detecting the simulated fixed effect *β* and also the accuracy of parameter estimation.

There are several parameters that determine the distribution of the simulated count data. To minimize any possible bias and to yield reasonable count values that are similar to real microbiome data, we randomly generated these parameters from wide ranges of values partially drawn from the real data described in the next section:

1. The values *T*_*i*_ are total reads, and *μ* is the overall mean. Thus, the values, log(*T*_*i*_) + *μ*, control the mean of simulated counts. We set *μ* = −7 and randomly sampled values, log(*T*_*i*_), from the range [7.1, 10.5]. In this case, log(*T*_*i*_) + *μ* falls in the range [0.1, 3.5], which yield similar counts as in the real microbiome data;
2. The shape parameter θ controls over-dispersion; we uniformly sample θ from the range [0.1, 5], which yield highly or moderate over-dispersed counts;
3. To evaluate false positive rates, the fixed effect *β* was set to be zero, and to evaluate empirical power, *β* was set to be low from [0.2, 0.35], or high from [0.4, 0.55];
4. To generate the random effects *b*_*k*_, we first sampled τ from the range [0.5, 1] and then *b*_*k*_ from N(0, τ^2^);
5. The correlation coefficient *ρ* was set to be weak from [−0.1, 0.1], positive from [0.5, 0.8], or negative from [−0.8, −0.5].

The ranges of all the parameters used in the simulation are summarized in Table 2.

**Table 2:** Parameter Ranges in Simulation Studies

Parameter	Range
log (T_i_) + μ	Unif (0.1, 3.5)
Shape parameter θ	Unif (0.1, 5)
Fixed effect β	0, Unif (0.2, 0.35), Unif (0.4, 0.55)
Standard deviation τ	Unif (0.5, 1)
Correlation ρ	Unif (−0.1, 0.1), Unif (0.5, 0.8), Unif (−0.8, −0.5)

For each combination of the parameters, the procedure was repeated 5000 times. Both empirical power and Type I error for testing the hypothesis H_0_: β = 0 were calculated under several significance (alpha) levels. We compared the proposed NBMMs with three existing methods:

1. The linear mixed model with the log transformation (LMM_log): $\log \frac{y_i+1}{T_i}={\beta}_0+{x}_i\beta +{z}_ib+{e}_i$, *b* ~ *N*_*K*_(0, *τ* ^2^ *I*), *e*_*i*_ ~ *N*(0, *σ* ^2^);
2. The linear mixed model with the arcsine square root transformation (LMM_arcsine): $\mathrm{arcsine}\left(\sqrt{\frac{y_i}{T_i}}\right)={\beta}_0+{x}_i\beta +{z}_ib+{e}_i$, *b* ~ *N*_*K*_(0, *τ* ^2^ *I*), *e*_*i*_ ~ *N*(0, *σ* ^2^);
3. The negative binomial model (NB): *y*_*i*_ ~ *NB*(*y*_*i*_|*μ*_*i*_, *θ*), log(*μ*_*i*_) = log(*T*_*i*_) + *β*_0_ + *x*_*i*_ *β*;
4. The linear model with the arcsine square root transformation (LM): $\arcsin \left(\sqrt{\frac{y_i}{T_i}}\right)={\beta}_0+{x}_i\beta +{e}_i$, *e*_*i*_ ~ *N*(0, *σ* ^2^).

#### Simulation results

**Fig. 1:**
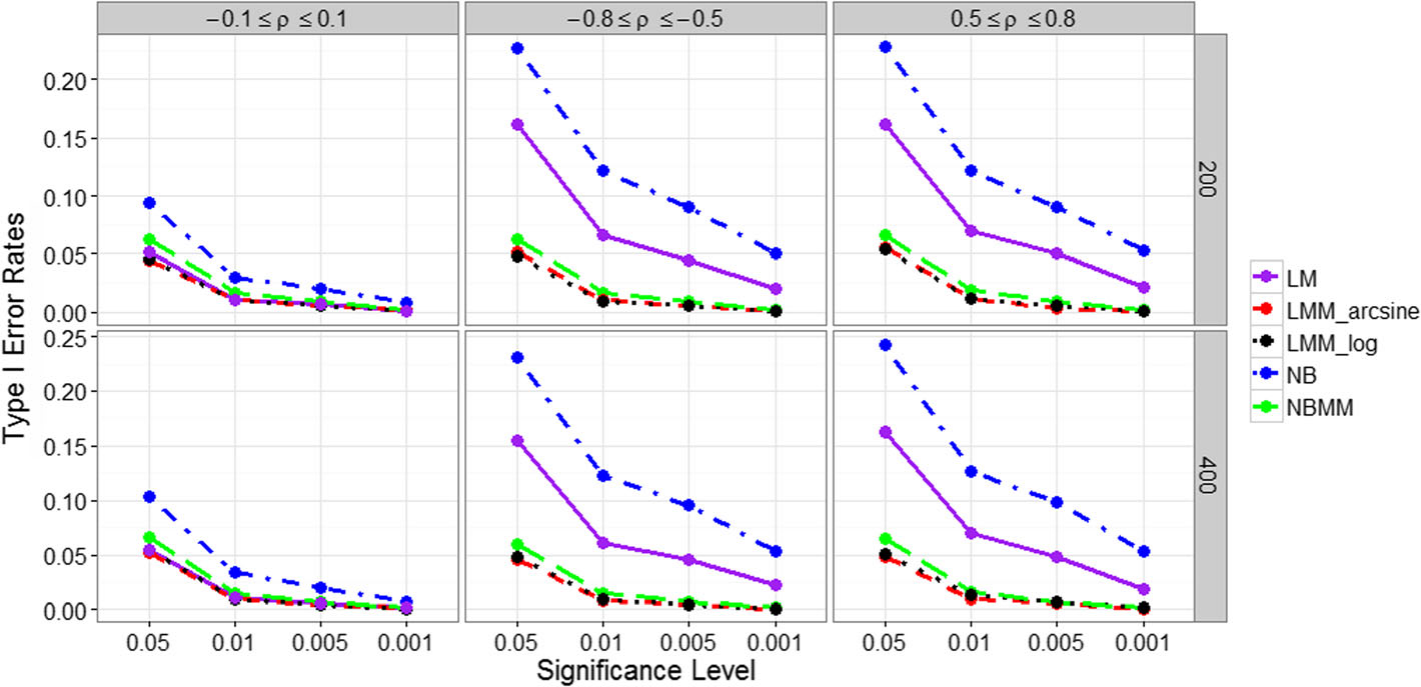
Type I error rates for the five methods in different simulation settings

Figure 1 displays Type I error rates for detecting the fixed effect under four significance levels for the four methods. We found that sample size *n* had minimal effects on Type I error. However, the correlation between the host variable and the random factor affected Type I error if the random factor was not included in the model. Under the weak correlation setting (i.e., ρ ϵ [−0.1, 0.1]), the four methods, NBMM, LMMs (LMM_arcsine and LMM_log) and LM, controlled Type I error under or close to the nominal level, however, NB had slightly inflated Type I error. In both positive and negative correlation settings (i.e., ρ ϵ [0.5, 0.8] or [−0.8, −0.5]), NB and LM had largely inflated Type I errors, however, NBMM and LMMs still resulted in well controlled Type I errors. This implies that ignoring the random effects can be misleading and can produce severely biased results.

**Fig. 2:**
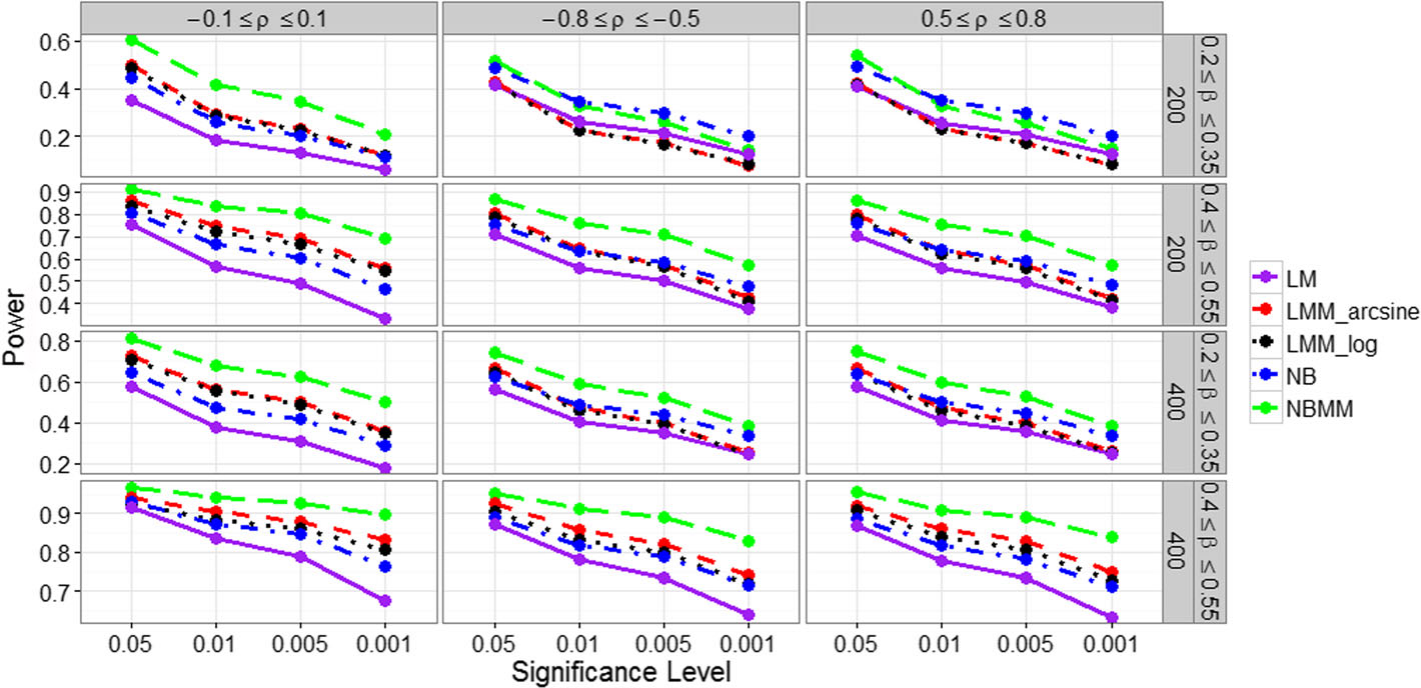
Empirical powers for the five methods in different simulation settings

Figure 2 shows empirical power for detecting the fixed effect under four significance levels for the four methods. As expected, the power was largely affected by the sample size and the effect size. However, the correlation between the host variable and the random factor had little influence on the empirical power. It can be clearly seen that the proposed method performed consistently much better than the other methods across all the scenarios. For most scenarios, LMMs were able to produce higher power than NB. Therefore, our NBMM that accounts for the dependence of samples and directly analyzes the generated count data produces increased power to detect the fixed effect of interest.

**Fig. 3:**
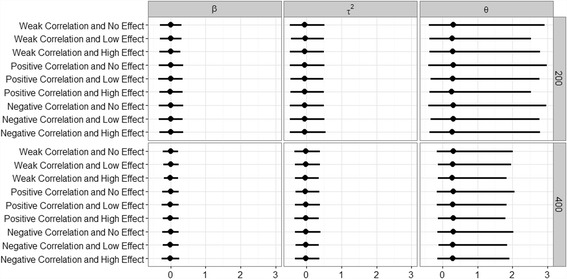
Differences between the estiamates and their simulated values for the parameters β, τ^2^, and θ in the proposed NBMM in different simulation settings. The points represent the average values and the lines represent the interval estimates

Figure 3 displays the differences between the estimates of three parameters, the fixed effect β, the variance τ^2^ and the shape parameter θ, and their simulated values, in our NBMM. It can be seen that the estimates of β and τ^2^ were very close to the corresponding simulated values under all the scenarios and the estimates of the shape parameter were slightly inflated. These results show that the proposed IWLS algorithm was able to provide accurate model fit. We found that models in which the estimates of the shape parameter were inflated usually gave larger residual variances σ^2^. This finding indicates that with the additional parameter σ^2^, our method can robustly deal with over-dispersion even if the shape parameter was not accurately estimated.

#### Application to mouse gut microbiome data

We applied our method to a robust mouse gut microbiome data set from the genetic analysis of Leamy et al. [39]. The population of 472 mice is the tenth generation of advanced intercross from an original cross of inbred C57BL/6 J (B6) female mice with male mice from a strain (HR) selected for a high level of voluntary wheel running. The 472 progeny G_10_ mice were generated from 45 G_9_ dams and 42 G_9_ sires, forming family structuring in the G_10_ progeny. The detailed protocol for mating can be found in Leamy et al. [39]. At 4 weeks of age, all G_10_ progeny mice were randomly allocated into two diet groups, one fed with high-fat diet, the other fed with control diet. At 8 weeks of age, the fecal pellets of mice were collected for DNA extraction and subsequent pyrosequencing. Composition of the microbiota was assessed by deep pyrosequencing of PCR products originating from the V1-V2 region of the 16S rRNA gene with bar-coded fusion primers containing Roche-454 A or B Titanium sequencing. 203 taxa were detected for the species level data. These species belong to 104 different genera, 45 families, 29 orders, 22 classes and 14 phyla. The median value of the total reads across all animals was 14170 and the standard deviation was 3422.

We used the proposed NBMM and two linear mixed models (LMMs) with the arcsine square root transformations and log (LMM_arcsine and LMM_log) to detect associations between taxa and high-fat diet. Since the maternal environment have a profound influence on the microbiota composition [11], we included dam indicators as a random factor in the NBMM and LMMs. In LMM_arcsine, we treated the arcsine square root transformed values, $\arcsin \left(\sqrt{\frac{y_i}{T_i}}\right)$, as normally distributed, where *y*_*i*_ is the microbiome count and *T*_*i*_ is the total sequence read for the *i*-th animal. In LMM_log, we treated the transformed values $\log \frac{y_i+1}{T_i}$ as normally distributed. These two LMMs performed similarly and thus only results of LMM_arcsine were shown in the following figure. Leamy et al. [39] also analyzed the associations between taxa and high-fat diet. However, their analyses compared estimates of alpha diversity in the microbiota across animals fed control or high-fat diets and used ANOVA to identify significant taxa not accounting for the dam effects.

**Fig. 4:**
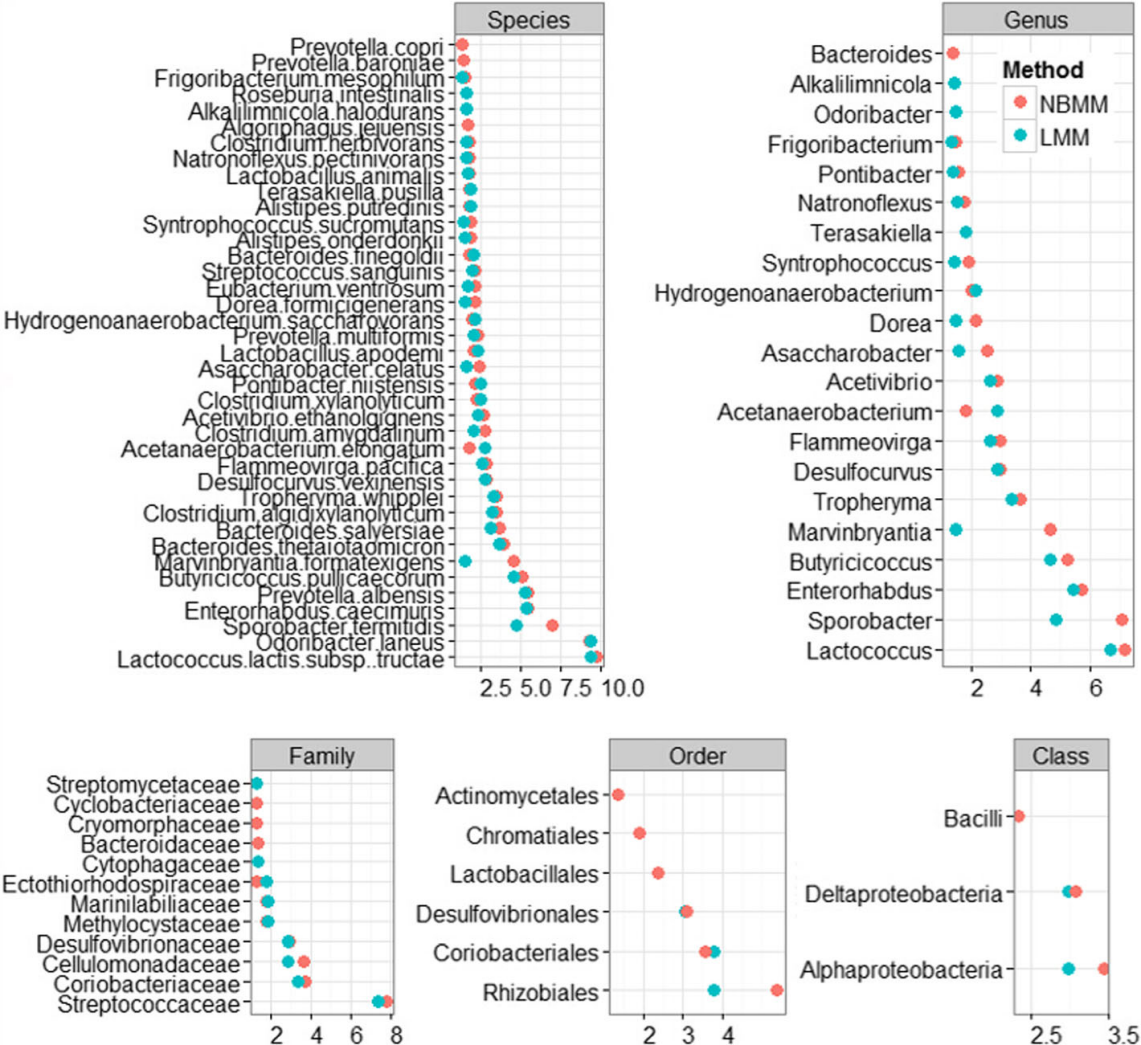
The analyses of NBMM and LMM with the arcsine square root transformation: minus log transformed p-values for the significant differentially abundant taxa at the 5% significance threshold between high fat diet and control diet groups for species, genus, family, order, and class levels

Figure 4 shows the significant features of the species, genus, family, order, class and phylum levels at the 5% significance threshold and their minus log transformed p-values for NBMM and LMM_arcsine. It can be seen that For NBMM and LMM_arcsine the identified significant features were mostly overlapped. However, the proposed NBMM method produced smaller p-values for most of the identified features, and detected more significant features than the LMM_arcsine. These results indicate that our NBMM approach is more powerful to detect significant features than the previously used LMMs in real data analysis.

The top significant genera include *Lactococcus, Sporobacter, Enterorhabdus, Marvinbryantia,* and *Butyricicoccus*. Some of the identified features are associated with metabolic health and have been previously reported in other studies. At the species level, *Marvinbryantia formatexigens* has been reported to decrease with increased casein levels in dietary intervention, and the genus *Marvinbryantia* is also believed to be associated with human health [51, 52]. *Eubacterium ventriosum* has been found to be shifted by high fat diet [53] and *Alistipes putredinis* has also been found to be of particular interest in its association with obesity [54]. The genus *Lactococcus* has been reported to decrease overtime in high-fat diet fed mice [51, 55]. The genus *Enterorhabdus* has been reported to be positive correlated with intrahepatic levels triacylglycerol concentrations and non-HDL plasma concentrations in mice or hamsters [56]. The genus *Butyricicoccus* has also been discovered to decrease in mice fed a high-fat diet [57]. Studies have also found that the species *Tropheryma whipplei*s is associated with acquired obesity [58, 59]. It is also worth noting that certain genera were only detected by NBMM, including *Bacteroides*, which has been reported as an important genus in high-fat fed animals gut microbiome to potentially act as Obesity-Associated Metabolic Parameters [51, 60–62].

## Discussion and conclusions

We have proposed a negative binomial mixed model to detect the associations between host clinical/environmental factors and the microbiome while accounting for sources of heterogeneity and dependence in microbiome measurements. Many microbiome studies collect samples with hierarchical, spatial, and temporal structures [33–38]. These properties have important implications in the analysis and interpretation of microbiome data. Our simulation studies illuminated the impact of such structuring on the data, showing that ignoring the correlation among samples can substantially inflate Type I error and reduce power for detecting the effects of host clinical/environmental factors, thus leading to biased and false inferences. Due to the lack of efficient statistical methods and programs, most previous studies used conventional linear mixed models (LMMs) by treating transformed relative abundance data as normally distributed response. Although useful in some situations, LMMs can be less powerful than the proposed method as shown in our simulation studies.

We applied our method to previously published data set of a genetic analysis to detect host genetic factors that control compositional featurs of the gut microbiome [39]. The goal of our analysis was to detect taxa that are significantly associated with the effect of high-fat diet, which was introduced as an environmental variable in the original study to examine G x E effects on microbiome composition. Many studies found that the maternal environment have a profound influence on the microbiota composition [11, 63–66]. Thus, it is necessary to incorporate the dam indictors as a random factor into the model to correct for possible counfounding effects. Our analysis identified several significant and biologically meaningful taxa that have been previously reported in other studies. Our NBMM method was able to detect more significant taxa and yield much smaller *p*-values than the LMMs, showing that the proposed method could be more powerful than the conventional LMMs in real data analysis.

The proposed NBMMs directly model microbiome counts generated by the 16S rRNA gene sequencing or the shotgun sequencing. Since most bioinformatics tools produce count data in microbiome studies, the proposed method has broad applications. For shotgun metagenomic data, some tools such as MetaPhlAn only output the relative abundances or proportions of the bacteria in the sample. Chen and Li [40] have recently developed zero-inflated Beta mixed-effects models to analyze the proportion data. Although we focus our analysis on microbiome studies, the proposed method are applicable to other similar types of sequence count data such as RNA-Seq. Most of the statistical methods and computer software for analyzing RNA-Seq data are developed based on negative binomial models [27, 67], but have not incorporated random effects. Our ability to deal with other types of sequence count data further broadens the biological impact of the proposed approach.

We have developed an IWLS algorithm to fit the proposed NBMMs by extending a commonly used procedure for fitting GLMs and GLMMs. [42, 44–46] The idea of the algorithm is to approximate the negative binomial likelihood given the shape parameter by a weighted normal likelihood and then to update the parameters by fitting a linear mixed model. This procedure for GLMs and GLMMs has been proved to be highly useful and efficient. Our extensive simulations and real data analysis show that our algorithm is stable and efficient.

The proposed NBMMs with the IWLS algorithm have several remarkable features. Due to the introduction of an additional parameter *σ* ^2^ to correct for over-dispersion, the proposed method can be robust and efficient to deal with over-dispersed data. Our approach takes advantage of the fitting procedure of LMMs to update the parameters, and hence can in principle incorporate all the features of LMMs into the NBMMs. Although we describe our method with a simple random effect, the proposed method can be applied to various patterned covariance structures for modeling special random effects [42, 43], for example, family, longitudinal, repeated measures or kinship structures. The assumption Var(e) = σ^2^I can be relaxed as described in Pinheiro and Bates [43], where they discuss extensions that allows us to model non-constant variances or special correlation structures. All these extensions will be incorporated into the proposed NBMMs. Microbiome data have the distinct characterstic of zero-inflation. The proposed NBMMs are not particularly designed to deal with zero-inflation, although we suggest it as a future work.

## Abbreviations

ANOVA: Analysis of variance
GLMMs: Generalized linear mixed models
GLMs: Generalized linear models
HDL: High-density lipoproteins
IWLS: Iterative Weighted Least Squares
LMMs: Linear mixed models
NB: Negative binomial
NBMMs: Negative binomial mixed models
